# Prevalence, characteristics, and survival of frontotemporal lobar degeneration syndromes

**DOI:** 10.1212/WNL.0000000000002638

**Published:** 2016-05-03

**Authors:** Ian T.S. Coyle-Gilchrist, Katrina M. Dick, Karalyn Patterson, Patricia Vázquez Rodríquez, Eileen Wehmann, Alicia Wilcox, Claire J. Lansdall, Kate E. Dawson, Julie Wiggins, Simon Mead, Carol Brayne, James B. Rowe

**Affiliations:** From the Department of Clinical Neurosciences (I.T.S.C.-G., K.P., P.V.R., A.W., C.J.L., K.E.D., J.W., J.B.R.) and Cambridge Institute of Public Health (C.B.), University of Cambridge; Dementia Research Centre (K.M.D.) and MRC Prion Unit and Department of Neurodegenerative Disease (S.M.), University College London, UK; and University Medical Center Eppendorf (E.W.), University Hamburg, Germany.

## Abstract

**Objectives:**

To estimate the lifetime risk, prevalence, incidence, and mortality of the principal clinical syndromes associated with frontotemporal lobar degeneration (FTLD) using revised diagnostic criteria and including intermediate clinical phenotypes.

**Methods:**

Multisource referral over 2 years to identify all diagnosed or suspected cases of frontotemporal dementia (FTD), progressive supranuclear palsy (PSP), or corticobasal syndrome (CBS) in 2 UK counties (population 1.69 million). Diagnostic confirmation used current consensus diagnostic criteria after interview and reexamination. Results were adjusted to the 2013 European standard population.

**Results:**

The prevalence of FTD, PSP, and CBS was 10.8/100,000. The incidence and mortality were very similar, at 1.61/100,000 and 1.56/100,000 person-years, respectively. The estimated lifetime risk is 1 in 742. Survival following diagnosis varied widely: from PSP 2.9 years to semantic variant FTD 9.1 years. Age-adjusted prevalence peaked between 65 and 69 years at 42.6/100,000: the age-adjusted prevalence for persons older than 65 years is double the prevalence for those between 40 and 64 years. Fifteen percent of those screened had a relevant genetic mutation.

**Conclusions:**

Key features of this study include the revised diagnostic criteria with improved specificity and sensitivity, an unrestricted age range, and simultaneous assessment of multiple FTLD syndromes. The prevalence of FTD, PSP, and CBS increases beyond 65 years, with frequent genetic causes. The time from onset to diagnosis and from diagnosis to death varies widely among syndromes, emphasizing the challenge and importance of accurate and timely diagnosis. A high index of suspicion for FTLD syndromes is required by clinicians, even for older patients.

Frontotemporal lobar degeneration (FTLD) causes diverse clinical syndromes including behavioral variant frontotemporal dementia (bvFTD), with or without motor neuron disease (MND); primary progressive aphasias (PPAs) (semantic variant [svPPA], nonfluent agrammatic variant [nfvPPA], and logopenic variant); progressive supranuclear palsy (PSP) (Steele-Richardson-Olszewski syndrome); and the corticobasal syndrome (CBS). These syndromes are common causes of young-onset dementia,^1,2^ but there are potential limitations to previous estimates of prevalence and incidence. First, the diagnostic criteria have been revised significantly in recent years^3–6^ with changes in specificity and sensitivity.^4^ For example, many patients who met former criteria for bvFTD had normal imaging and minimal progression.^7^ These “phenocopy” cases are now excluded. Second, intermediate phenotypes of uncertain nosologic status lay outside former diagnostic categories, e.g., the overlap between PSP and CBS.^5,8^ Furthermore, a patient's syndrome may evolve to another.^9,10^ As a consequence, the sum of prevalence estimates of single disorders might not accurately reflect their overall prevalence. Third, previous studies used widely varying methods, often with cumulative rather than point prevalence and different age ranges. This hinders comparisons across studies.^2^

To address these limitations, we prospectively recruited all cases over 2 years from 2 UK counties (population 1.69 million) using multisource identification from primary, secondary, and tertiary care, self-referral, and relevant patient charities. We reassessed them and applied the new diagnostic criteria with the goal of achieving a more accurate estimate of the prevalence and incidence of the major FTLD-associated syndromes.

## METHODS

The PiPPIN (Pick's Disease and Progressive Supranuclear Palsy: Prevalence and Incidence) Study was approved by Cambridge's research ethics committee. The catchment area included Cambridgeshire and Norfolk in the East of England. Their combined population was 1.69 million according to the 2013 UK Office for National Statistics midyear estimate,^11^ covering urban and rural populations with a full socioeconomic range. The area subdivides into 13 local authority districts with populations of 85,398 to 188,373.^11^ We sought identification of all cases with a reference diagnosis between January 1, 2013, and December 31, 2014.

Multiple sources of case identification were used, including regional specialist clinics for frontotemporal dementia (FTD), other disorders of movement and cognition, and early dementia. These clinics receive referrals from primary, secondary, and tertiary care services for dementia, Parkinson disease and related disorders, MND, adult neurology, medicine for the elderly, adult- and old-age psychiatry; memory clinics; and regional community-based specialist nurses for Parkinson disease, young-onset dementia, and dementia. Referral sources were contacted in person, by letter, and by e-mail before and during the study. Direct referrals were accepted from clinical research networks including the National Institute for Health Research Clinical Research Network Dementias and Neurodegeneration Speciality and the West Anglia Clinical Research Network. We included patients notified to us by self-referral, local newspaper advertisements for the PiPPIN Study, and letters of invitation to members of local and national charities (the UK FTD support group and PSP Association).

To identify people who were no longer under review, we searched our clinic databases for cases with a relevant diagnosis since 2003. We confirmed survival and address by clinical records. To maintain awareness of PiPPIN and promote case notification of patients, the study team made frequent presentations to relevant national and regional conferences, meetings, research groups, support groups, and charities.

Referring services were required to record consent for case notification. Patients assessed within the study (including notes review) provided additional written informed consent. Patients who lacked mental capacity to consent to research participation under UK law were eligible for inclusion, and we adopted the “consultee process” as set out by the Mental Capacity Act (2005).

For patients who were willing to be reviewed by the study team, a neurologist applied the revised diagnostic criteria based on clinical interview, physical examination, and relevant tests including brain imaging. For cases that were unable or unwilling to be assessed in person, we accessed existing medical records.

Participants were invited to a detailed clinical assessment either at the study center or their home, residential home, or nursing home. This included (1) semistructured interviews of patient and carer for clinical history and demographic data; (2) structured patient and carer assessment of symptoms and severity; (3) structured assessment of speech, language, and cognition; (4) neurologic examination; and (5) MRI of the brain. Results of investigations during clinical diagnosis were accessed from the patient's medical records including neuropsychological assessments, neuroimaging, and genetic testing. Inability to travel, or residence in a care home, did not preclude participation in any aspect of the study, except MRI. For patients unable or unwilling to undergo parts of the assessment, a pragmatic approach was taken prioritizing diagnostic accuracy. Forty-six patients consented to additional genetic screening for 17 known genetic causes of FTLD and non-FTLD dementia.^12^

The diagnostic criteria for bvFTD,^4^ PPA syndromes,^3^ PSP,^6^ and CBS^5^ were applied. We present the dominant syndrome or phenotype using these criteria. To be inclusive of all cases within the spectrum of FTLD-associated disorders, special consideration is required when applying the criteria to cases that lie at a boundary between 2 categories, or who present with an overlap of clinical features. For example, a hypothetical patient may present with an atypical svPPA, with their principal complaint being typical of the language disorder but with predominant behavioral disturbance under observation. Such a patient may lie outside of the svPPA criteria^3^ but also not meet bvFTD criteria in view of the level of semantic deficits and absence of other bvFTD features. Excluding such a patient would underestimate prevalence of FTLD-associated syndromes. Conversely, another patient might meet criteria for CBS-NAV^5^ but also nfvPPA.^3^ They should clearly only be counted once when estimating the aggregated prevalence of FTLD-associated syndromes. Where there was diagnostic ambiguity, a second neurologist reviewed the case and a consensus was reached, based on the principal syndromic features.

*Age at onset* was the age of the patient when the earliest symptom of dementia was noted by the patient or carers. *Age at diagnosis* was defined as the age at which a neurodegenerative disorder was first considered as a likely cause of the patient's symptoms by a specialist. We also recorded the date at which a specific FTLD-associated diagnosis was first considered. *Prevalent cases* were defined as those alive on January 1, 2014. *Incident cases* were defined as those first diagnosed during the study period. *Survival* in this report refers to the duration from age at onset to death, of patients who died during the study period.

Prevalence rates were calculated as the number of prevalent cases divided by the population of the catchment area using the UK Census 2013 midyear estimates.^11^ To identify regional differences in case identification, prevalence rates were also calculated for each local authority. Incidence was calculated by dividing the total number of incident cases by the total number of person-years for the catchment area population over 2 years. Standardized rates were calculated using the Revised European Standard Population 2013 (ESP2013). Age- and sex-standardized lifetime risk was calculated using the current probability method.^13,14^ First, age-specific prevalence, incidence, and mortality rates were standardized to the ESP2013. Using incidence and death rates from our data, we calculated the risk of developing disease and of dying of it within specific age ranges. We then adjusted for the risk of dying of other causes (using census all-cause mortality) to give age-specific current probability of developing disease. The sum of current probabilities for all age groups gives the lifetime risk, i.e., the probability of an individual developing FTLD during their life adjusted for the risk of dying of other causes first.^15^

## RESULTS

Two hundred thirty-four patients were assessed; 176 were referred via clinical services, 52 from clinical and research database searches. One hundred ninety-seven patients were reviewed in person by the study team (84.2%). Two hundred four cases of FTLD-associated syndromes were identified in the catchment area over the study period; 30 patients were rejected after review. Of the 204 patients, 167 patients (81.7%) were seen by the study team. Clinical records of consultant neurologist or psychiatrist assessments were available in all of the remaining cases. Detailed information sufficient to apply the diagnostic criteria in full was available for 200 cases (98.0%). The patients with limited information included 2 with PSP and 2 with FTD-MND. Table 1 shows the mean age, clinical features, and years since symptom onset at the time of assessment. The bvFTD group includes 8 cases with clinical features of MND (19%, table 1).

**Table 1:**
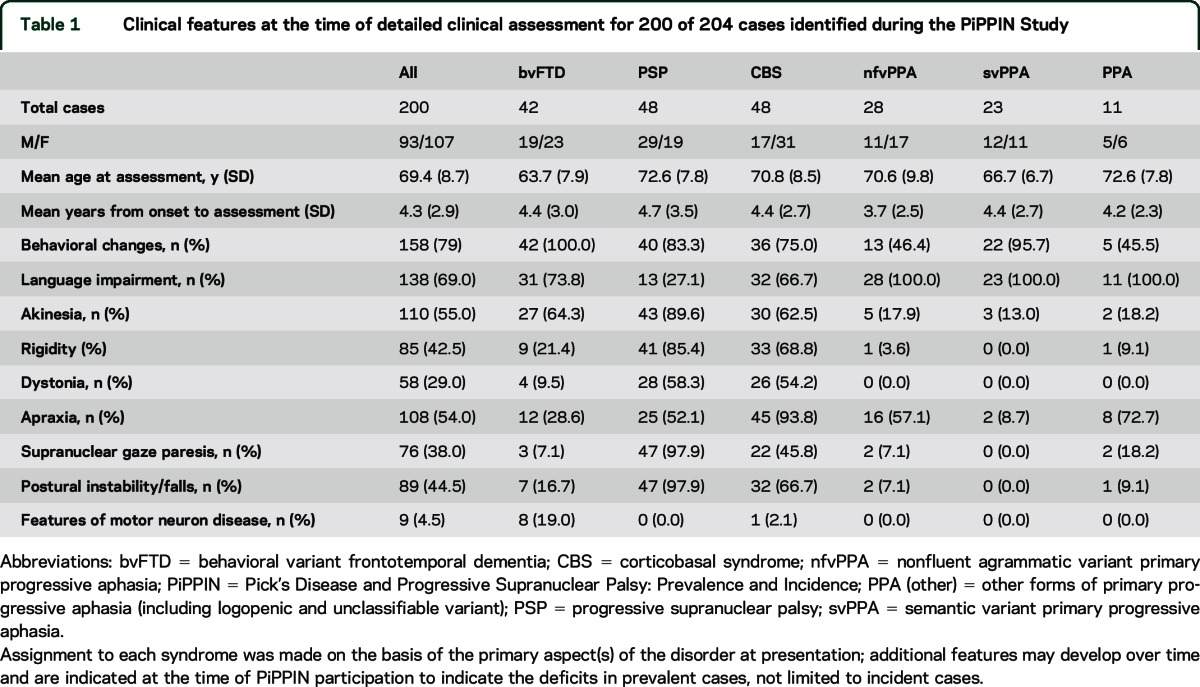
Clinical features at the time of detailed clinical assessment for 200 of 204 cases identified during the PiPPIN Study

One hundred eighty-two patients were alive on January 1, 2014 (89 men, 93 women), giving a crude prevalence of 10.77/100,000. The European-standardized prevalence (95% confidence intervals) was as follows: men 10.93/100,000 (8.66–13.20), women 10.76/100,000 (8.57–12.95), and sex-standardized 10.84 (9.27–12.42).

**Figure 1:**
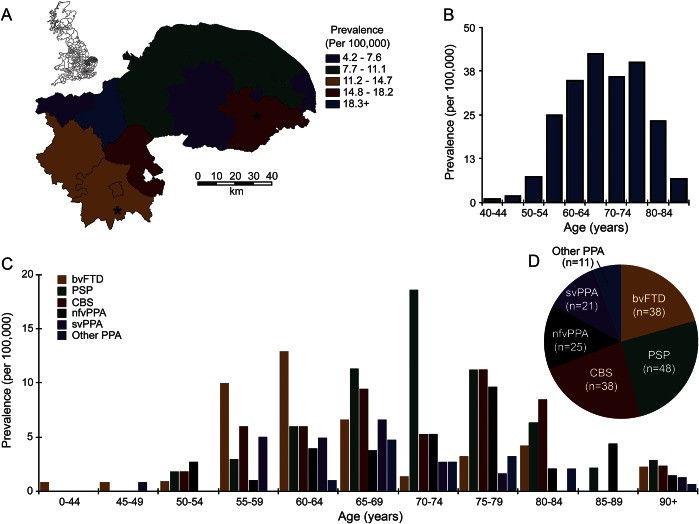
Prevalence of FTLD-associated syndromes (A) The local authorities in England, with enlargement of the PiPPIN catchment area in the East of England. The asterisks mark the 2 cities (Norfolk and Cambridge) within the catchment area. The color code indicates crude prevalence rates of FTLD-associated clinical syndromes for each local authority. (B) Crude prevalence rates for the major FTLD-associated syndromes by age and (C) by age and syndrome. (D) Total number of prevalence cases for each syndrome. bvFTD = behavioral variant frontotemporal dementia; CBS = corticobasal syndrome; FTLD = frontotemporal lobar degeneration; nfvPPA = nonfluent agrammatic variant primary progressive aphasia; other PPA = other primary progressive aphasia; PiPPIN = Pick's Disease and Progressive Supranuclear Palsy: Prevalence and Incidence; PSP = progressive supranuclear palsy; svPPA = semantic variant primary progressive aphasia. (A) Contains National Statistics data © Crown copyright and database right [2013]; contains Ordnance Survey data © Crown copyright and database right [2013].

Figure 1 shows prevalence rates by local authority boundaries, age of diagnosis, and syndrome. Prevalence rates by local authority ranged from 4.09 to 21.7/100,000 but were not driven by proximity to the major cities or study center.

The youngest age at onset was 41 years. The ESP2013 age- and sex-standardized prevalence for the age range 40–64 was 13.05 (10.01–16.08) and 33.20 (27.02–39.37) for those older than 65 years. One-way analysis of variance of age at diagnosis indicated a significant effect of diagnosis (*F*_5,181_ = 4.89, *p* < 0.001). Post hoc comparisons (unequal variances, Bonferroni correction) confirmed significant differences between PSP and bvFTD (*p* < 0.005), other PPA and bvFTD (*p* < 0.01), and PSP vs svPPA (*p* < 0.05).

**Figure 2:**
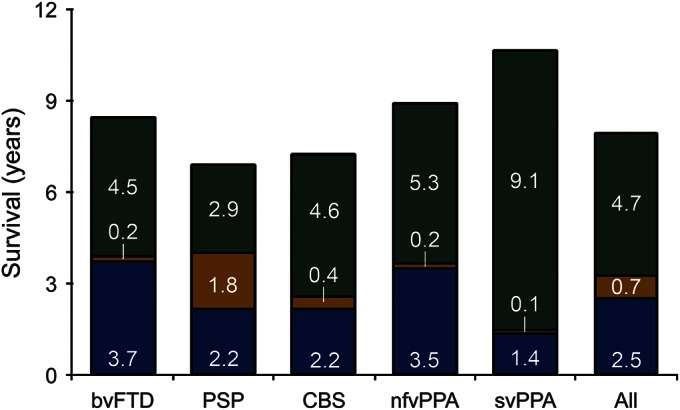
Survival with FTLD-associated syndromes Mean duration from the onset of symptoms to the diagnosis of a neurodegenerative disorder (blue); to the specific diagnosis of an FTLD-associated syndrome (gold); and to death (green) for all 60 patients who have died since the start of the PiPPIN Study. Inserted figures indicate the mean duration of each phase for each syndrome and all patients combined. bvFTD = behavioral variant frontotemporal dementia; CBS = corticobasal syndrome; FTLD = frontotemporal lobar degeneration; nfvPPA = nonfluent agrammatic variant primary progressive aphasia; PSP = progressive supranuclear palsy; svPPA = semantic variant primary progressive aphasia.

Age at onset and neurodegenerative and FTLD-associated syndrome diagnoses were available for 193 patients (95%). Missing data were substituted by the mean within syndrome. Fifty-four patients (23 men, 31 women) died during the assessment period, giving an age-adjusted all-cause mortality from FTLD of 1.57 (1.15–1.99) per 100,000 person-years. By April 31, 2015, 6 additional patients had died. Figure 2 shows the length of symptomatic disease split by time before a neurodegenerative diagnosis, FTLD diagnosis, and death for the 60 deceased patients.

**Figure 3:**
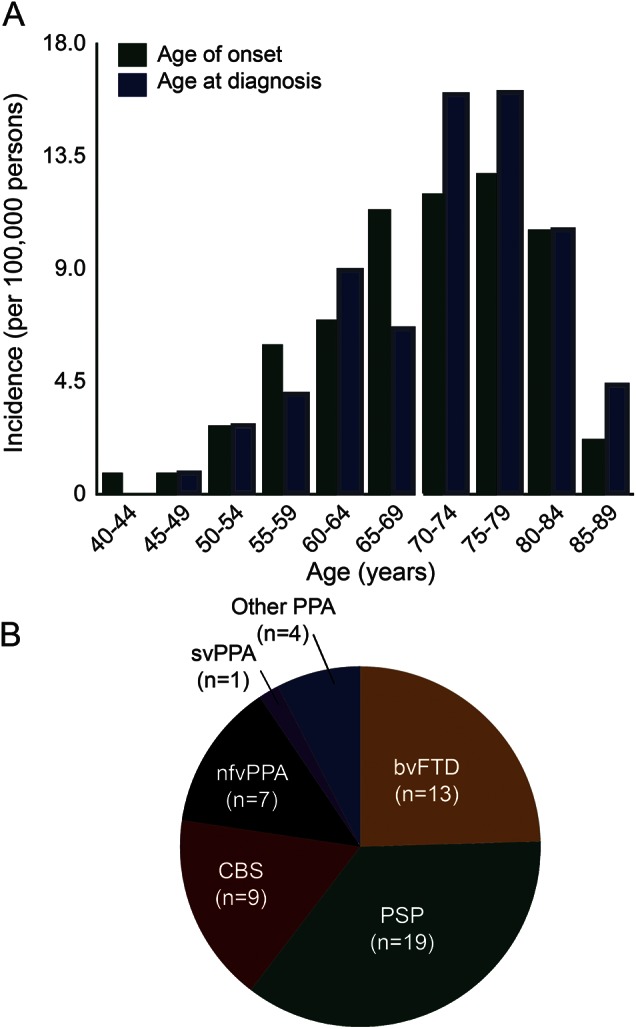
Incidence of FTLD-associated syndromes (A) Incidence of FTLD-associated syndromes by age at onset (green bars) and by age at diagnosis (blue bars). (B) Distribution of incident cases by clinical syndrome (n = 53). bvFTD = behavioral variant frontotemporal dementia; CBS = corticobasal syndrome; FTLD = frontotemporal lobar degeneration; nfvPPA = nonfluent agrammatic variant primary progressive aphasia; other PPA = other primary progressive aphasia logopenic variant (n = 2) and unclassifiable (n = 2); PSP = progressive supranuclear palsy; svPPA = semantic variant primary progressive aphasia.

Fifty-three incident cases were identified (24 men, 29 women), giving crude and standardized incidence rates of 1.57 and 1.61 (1.14–1.99) per 100,000 person-years, respectively. Figure 3 shows the crude incidence rates by age at onset and neurodegenerative diagnosis across all syndromes by age group. The lifetime risk (standardized for age and sex) was 1 in 742.

Forty-six patients (23%) underwent genetic screening (11 svPPA, 11 nfvPPA, 4 PPA [other], 17 bvFTD, 3 CBS, 1 PSP). Seven patients (15%) carried a relevant mutation (table e-1 on the *Neurology*® Web site at Neurology.org).

## DISCUSSION

We combined point-prevalence methods with an unrestricted age range and the 2011/2013 revised diagnostic criteria for major FTLD-associated clinical syndromes. We did not restrict our analysis to young-onset cases. The prevalence of all disorders taken together was 10.84/100,000 with similar prevalence of bvFTD, PSP and CBS, and PPA (all subtypes). The estimated lifetime risk of one of these disorders is 1 in 742.

The overall prevalence estimate is in line with previous studies, including those with restricted age ranges^1,16–20^ and/or selected syndromes.^2,17,19–24^ The data indicate that overall prevalence increases beyond age 65: the prevalence among those older than 65 years was more than double that of those aged 40 to 64 years. The bvFTD had the youngest peak age at diagnosis (peak 60–64 years, median age at diagnosis 63), with later peak ages at diagnosis for PSP (70–74 years) and CBS and nfvPPA (75–79 years).

Many previous studies have been restricted to individual syndromes. For example, when estimating the prevalence of FTD, some studies included PPA^1,19–21,25–27^ while others restricted their estimates to bvFTD,^16–18^ limiting ability to directly compare between them. For FTD (not including PSP/corticobasal degeneration), the UK prevalence was previously reported as 15.1/100,000 (8.4–27.0) in the 45 to 64 age group^1^ with an incidence of 1.3/100,000 (2.0–5.7) person-years.^25^ The diagnostic criteria used in these studies may have included nonprogressive “phenocopies.”^7^ We identified (and excluded) 2 such cases during screening. The identification of pathologic expansions of *C9ORF72*^28^ has renewed interest in these cases, but both cases were negative for *C9ORF72*. However, we identified (and included) one slowly progressive case who at first presentation had normal brain imaging and would not initially have met current criteria for probable bvFTD. He subsequently developed generalized atrophy and was identified as carrying a pathologic expansion of *C9ORF72*.

We included PSP and CBS in the study, as syndromes associated with FTLD. Although they often present with a movement disorder, they may have prominent cognitive and behavioral features in a third of cases,^29^ overlapping with other FTLD-associated syndromes,^8–10,30^ especially nfvFTD and bvFTD (see table 1). The overlap tends to increase with time.^9,10^ Epidemiologic studies of PSP have mainly used case notification to specialist centers or reviewed cases of parkinsonism. With reexamination of suspected cases, prevalence was estimated to be 0.15/100,000 and incidence 0.3–0.4/100,000.^22^ A second study^23^ used a computerized search of terms relating to parkinsonism and clinical reevaluation to estimate prevalence of 6.4/100,000 (2.3–10.6). A third study^24^ used both methods to estimate prevalences of 0.3 and 5.0/100,000.

There are limited and divergent data on the epidemiology of CBS. For example, one case was identified from 534 incident cases of parkinsonism^31^ while none were identified among an urban population of 121,628.^23^ A limiting factor for studies of CBS is diagnostic uncertainty. Fewer than two-thirds of cases with clinical CBS have pathologic features of corticobasal degeneration and vice versa.^32^ We used the revised diagnostic criteria,^5^ which aim to improve sensitivity, and did not restrict our cohort to movement disorder clinics or incident cases of parkinsonism. By selecting cases based on both motor and nonmotor aspects of their illness, we identified substantially more cases than previous studies.^23,31^

Despite revised criteria, diagnostic difficulties remain that will affect epidemiologic research, especially for studies that focus on one syndrome rather than the broader spectrum of FTLD-associated syndromes. Furthermore, the nosology and nomenclature of FTLD syndromes have changed many times^9^ and may continue to evolve. Although categorical decisions are required for diagnosis, the boundaries between FTLD-associated syndromes become indistinct with time. For example, 95% of patients with svPPA had developed behavioral changes, and most patients with bvFTD subsequently developed language impairment. Language impairments were common in CBS (66.7%), behavioral changes in PSP (83.3%), and motor features commonly emerged in all FTD subtypes. Our inclusive approach does not undermine the importance of diagnostic classification but emphasizes the heterogeneous and progressive nature of these disorders. The data demonstrate the need to account for transitional and intermediate clinical phenotypes in epidemiologic estimates and in consideration of clinical trials.

Within the spectrum of FTLD, the subtypes are each associated with more than one clinical syndrome. We cannot speak to the pathology of our cases except in the genetic cases and refer the reader to published studies of clinicopathologic correlations.^32–37^

We sought to identify and examine all cases where FTLD was considered, but cases in which it was not suspected will have been missed. The majority of cases were known to tertiary clinical services; however, relying solely on this route would have missed nearly a quarter of cases. Sources of referral were not independent, and UK data protection and confidentiality laws prevent identification of any cases that were not referred and this limits analysis of potential referral bias. From discussions with referrers in regional meetings, we are aware of only one patient who initially refused to be included in the study, suggesting good ascertainment of known cases of FTLD. However, in a community-based study of aging,^38^ 3 of 456 postmortem cases had evidence of PSP pathology. All 3 had symptoms that could be retrospectively attributed to PSP but none were diagnosed during life (Brayne, unpublished data, 2014). Hence, the true prevalence and incidence of FTLD *pathology* may be substantially higher than the prevalence and incidence of diagnosed cases. Similarly, the decrease in prevalence among older age groups may be attributable to reduced index of suspicion for FTLD syndromes. Rising rates of non-FTLD neurodegeneration (e.g., Alzheimer disease) may overshadow less common conditions such as FTLD or (in the case of dual pathology) alter the clinical phenotype so that an FTLD diagnosis is not recognized.

We confirmed all identified cases were alive and resident within the catchment area during the study. By liaising with national charities and clinical services beyond the PiPPIN catchment area, we aimed to identify any cases not previously known to our services who migrated into the area.

We did not include cases of MND without FTD although we are aware that some MND cases will have mild cognitive impairments. MND has a lifetime risk of approximately 1 in 400.^15^ Including MND cases with only mild cognitive impairment would overestimate the prevalence of symptomatic FTD syndromes. Eight (19%) of our bvFTD cases had clinical features of MND. The case of CBS with MND features is notable, possibly reflecting the range of pathologic causes of CBS.^32^

It should also be noted that screening for genetic mutations was not performed randomly. The screened cohort was dominated by nonmotor syndromes (PPA or bvFTD). Among this subgroup, the frequency of mutations identified is similar to previous studies since the discovery of *C9ORF72*.^39^ The finding of relevant mutations in 2 patients with typically sporadic syndromes (svPPA and CBS) illustrates the challenges to clinical segregation of FTLD. Three of 7 patients carrying mutations were diagnosed before the age of 60. It is conceivable that the presence of genetic mutations or additional features (such as MND) may influence factors such as age at onset or survival. However, the small numbers reported here preclude sufficiently powered comparisons.

The incidence of 1.61/100,000 person-years is similar to the mortality (1.56/100,000 person-years). Assuming constant prevalence, mortality serves as an additional surrogate estimate of incidence, suggesting that the study achieved “steady state” of referrals of incident cases. We report standardized prevalence and incidence estimates across all ages rather than report peak rates in the highest risk groups to allow comparison with other studies and other conditions. Time from onset to diagnosis and subsequent survival varied between syndromes. This may reflect different indices of suspicion for an underlying neurologic condition for different presentations or common misdiagnosis as a primary psychiatric disorder.

We have shown that the revised diagnostic criteria for FTLD-associated syndromes can be applied jointly in a multisource epidemiologic study without restriction to young-onset cases or single syndromes. The resulting prevalence, incidence, and survival data will allow better generalization of results from clinical, genetic, and pathologic studies of FTLD. Furthermore, they enable unbiased interventional studies with reference to the whole population of affected patients including sporadic cases and those older than 65 years.

## Supplementary Material

Data Supplement

## GLOSSARY

bvFTD: behavioral variant frontotemporal dementia
CBS: corticobasal syndrome
ESP2013: European Standard Population 2013
FTD: frontotemporal dementia
FTLD: frontotemporal lobar degeneration
MND: motor neuron disease
nfvPPA: nonfluent agrammatic variant primary progressive aphasia
PiPPIN: Pick's Disease and Progressive Supranuclear Palsy: Prevalence and Incidence
PPA: primary progressive aphasia
PSP: progressive supranuclear palsy
svPPA: semantic variant primary progressive aphasia
