# Supplementary material for: Prevalence, characteristics, and survival of frontotemporal lobar degeneration syndromes
Source: Neurology. 2016 May 3;86(18):1736–43. doi: 10.1212/WNL.0000000000002638 (PMC4854589; doi:10.1212/WNL.0000000000002638)
Supplement: Data Supplement [file supp_86_18_1736__index.html]

Data Supplement 

# Prevalence, characteristics, and survival of frontotemporal lobar degeneration syndromes

## Data Supplement

One table; one PDF file.

**Neurology® data supplements are not copyedited before publication. Published editorials and translations have been copyedited.  
 © 2016 American Academy of Neurology.  
  
 Files in this Data Supplement:**

- Table e-1 - PDF file
