# Supplementary material for: Prevalence, characteristics, and survival of frontotemporal lobar degeneration syndromes
Source: Neurology. 2016 May 3;86(18):1736–43. doi: 10.1212/WNL.0000000000002638 (PMC4854589; doi:10.1212/WNL.0000000000002638)
Supplement: Data Supplement [file supp_WNL.0000000000002638_Table_e-1.pdf]

Mutation	Syndrome	Age at Onset	Age at Diagnosis	Age at Assessment	Presenting Clinical Features	Additional Features at PiPPIN Assessment
PGRN c.385_388 delAGTC	navPPA	55	57	60	Progressive speech disturbance, word substitutions, phonological errors, impaired repetition and comprehension of complex tasks, orobuccal apraxia. MRI: left frontotemporal atrophy.	Rigid fixed routine, inappropriate giggling, jargon aphasia with phonological errors
MAPT c.-46G>A	lvPPA	69	71	72	Progressive speech difficulty, non fluent stuttering speech, phonological errors, length dependent impaired repetition. MRI: Global atrophy worst left temporo-parietally.	Mild episodic memory, visuospatial deficits, mild limb dyspraxia, myoclonus
TARDBP c.1147A>G	bvFTD	49	56	58	Progressive behavioural change, apathy, reduced empathy, fixed daily routine. MRI: Bilateral anterior temporal atrophy.	Semantic deficits, weakness and wasting of leg and paraspinal muscles, EMG confirmation of MND
MAPT c.1007A>G	svPPA	62	63	67	Isolated difficulty reading and understanding meaning of words. MRI: Bilateral anterior temporal atrophy worst on left.	Behavioural changes, disinhibition, apathy, fixed routine and loss of empathy
C9ORF72	bvFTD	57	57	60	Dysexecutive, apathetic syndrome, social withdrawal reduced empathy. MRI: global atrophy, worst frontally	Marked apathy, mute, mobile but with falls, no signs of motor neuron disease.

TREM2<br><br>c.140G>A	CBS-NAV	61	63	N/A*	Word finding and articulatory impairments. Agrammatic non fluent speech, mild asymmetric bradykinesia, MRI: biparietal and right temporal atrophy	*Marked behavioural disturbance, aggression, dyskinesia, dystonia, grasping behaviour, supranuclear gaze palsy, myoclonus.
C9ORF72	bvFTD	64	65	N/A*	Prominent behavioural change, disinhibition, dysexecutive cognitive profile. MRI: Within normal limits.	*Extrapyramidal syndrome (attributable to medication), falls. Normal DAT scan. MRI: Mild generalized atrophy

Supplementary Table e-1. Clinical and imaging features of the seven patients with an identified relevant mutation. *These two patients were not re assessed in person as part of the PiPPIN study. The clinical features are based on their clinical records subsequent to first presentation. PGRN-Progranulin, MAPT-Microtubule Associated Protein Tau, TARDBP –Transactive response DNA binding protein, C9ORF72 –Chromosome 9 Open Reading Frame 72, TREM2-Triggering Receptor Expressed on Myeloid Cells 2.
